# Herpes Zoster and Diabetes Mellitus: A Review

**DOI:** 10.1007/s13300-018-0394-4

**Published:** 2018-03-08

**Authors:** Marianthi Papagianni, Symeon Metallidis, Konstantinos Tziomalos

**Affiliations:** 1 First Department of Internal Medicine, Medical School, Aristotle University of Thessaloniki, Thessaloniki, Greece; 2 First Propedeutic Department of Internal Medicine, Medical School, Aristotle University of Thessaloniki, Thessaloniki, Greece

**Keywords:** Adjuvanted vaccine, Diabetes mellitus, Herpes zoster, Live attenuated vaccine, Post-herpetic neuralgia, Vaccination

## Abstract

Accumulating evidence suggests that diabetes mellitus (DM) represents an important risk factor for both herpes zoster and post-herpetic neuralgia. Moreover, post-herpetic neuralgia appears to be more severe and persistent in diabetic patients. On the other hand, a novel vaccine against varicella-zoster virus (VZV) was recently introduced in clinical practice. Given the increased risk and severity of herpes zoster infection in patients with DM, this vaccine might be useful in this population. However, there are limited data regarding the efficacy and safety of vaccination against herpes zoster in the diabetic population. The aim of the present review is to discuss the incidence and consequences of herpes zoster infection in DM and to comment on the role of vaccination against VZV in these patients.

## Introduction

Herpes zoster occurs after the reactivation of a previous infection of varicella zoster virus (VZV). The sensory ganglia appear to be the first infected during the primary exposure to VZV. Usually, a latency period of several years follows and then replication of the virus occurs. Subsequently, VZV travels along the affected sensory nerves to the skin and induces the characteristic vesicular rash accompanied by pain, both of which follow a dermatomal pattern [1–3]. Complications of herpes zoster involve the motor neuron and the central nervous system and include post-herpetic neuralgia, encephalitis, myelitis, and cranial and peripheral nerve palsies. Post-herpetic neuralgia, defined as persistent pain after the resolution of the rash, is probably the most distressing and challenging complication of herpes zoster. It can persist for years and may substantially impair the quality of life [4, 5].

The latency period of VZV depends on several factors, which reflect host-virus interactions. Aging and immunosuppression appear to play an important role [3, 6]. The incidence of herpes zoster and severity of post-infection complications increase with age [7]. Moreover, hospitalization rates and mortality also rise with age, with the majority of the cases occurring in adults ≥ 50  years old. Demographic changes and population aging are anticipated to increase the global burden of the disease [8].

Patients with diabetes mellitus (DM) are more susceptible to several infections because of impaired innate and adaptive immunity [9]. Cell-mediated immunity, phagocytosis and opsonization are attenuated in this population [10, 11]. In addition, patients with DM exhibit an imbalance of T cell homeostasis involving an expansion of CD4^+^CD28^null^ T cells and a reduction in CD4^+^CD25^+^Foxp3^+^ regulatory T cells, which also appears to predispose to VZV reactivation [12]. Several recent studies suggest that DM represents an important risk factor for both herpes zoster and post-herpetic neuralgia [13, 14]. In addition, DM was reported to increase the severity of the clinical course of herpes zoster [15]. On the other hand, two vaccines against VZV have been recently introduced in clinical practice [16]. Given the increased risk and severity of herpes zoster infection in patients with DM, these vaccines might be particularly beneficial in this population.

The aim of the present review is to discuss the incidence and consequences of herpes zoster infection in patients with DM and to comment on the role of vaccination against VZV in this population. This article is based on previously conducted studies and does not contain any studies with human participants or animals performed by any of the authors.

## Incidence of Herpes Zoster Infection in Patients with DM

Several studies showed that the incidence of herpes zoster infection is higher in patients with type 2 DM (T2DM) than in age-matched controls [13, 17–22]. In a recent meta-analysis of 62 studies, T2DM was independently associated with increased risk for herpes zoster infection (relative risk 1.30, 95% confidence interval 1.17–1.45) [14]. Importantly, it has been estimated that approximately 13% of cases of herpes zoster infection occur in patients with T2DM [19]. Moreover, undiagnosed T2DM is frequent in patients with herpes zoster infection, suggesting that these patients should be evaluated for the presence of T2DM [23].

Limited data suggest that type 1 DM (T1DM) is also a risk factor for herpes zoster infection [24, 25]. Interestingly, a recent study showed that T1DM might increase the risk for herpes zoster infection more than T2DM [24].

Cell-mediated immunity against VZV appears to be less potent in diabetic patients compared with non-diabetic subjects, and this might explain the increased risk of herpes zoster infection in this population [26]. Interestingly, glycemic control does not correlate with the severity of impairment of cell-mediated immunity [26]. In contrast, humoral immunity against VZV does not appear to be affected by DM [26].

In accordance with patients without DM, the incidence of herpes zoster increases with age and is also higher in women with DM than in men [27]. Interestingly, DM appears to increase the risk for herpes zoster infection more in the elderly than in younger patients [20]. Moreover, patients with micro- and macrovascular complications of DM are also at higher risk for herpes zoster than patients without complications [18, 24]. In addition, treatment with thiazolidinediones, alpha-glucosidase inhibitors, dipeptidyl peptidase-4 inhibitors (DPP-4) and insulin appears to increase the risk of herpes zoster, whereas metformin and sulphonylureas do not appear to affect this risk [18, 28]. The association between DPP-4 inhibitors and increased incidence of herpes zoster might be due to the inhibition of CD26 by these agents. Indeed, CD26 has intrinsic DPP-4 activity but is also present on the surface of T lymphocytes, B lymphocytes and macrophages and can cause T cell activation and proliferation [29]. Therefore, inhibition of CD26 by DPP-4 inhibitors might attenuate cell-mediated immunity [30].

## Consequences of Herpes Zoster Infection in Patients with T2DM

Post-herpetic neuralgia develops more frequently in patients with DM [13, 31–33]. In a recent large retrospective study (*n* = 420,515 cases of herpes zoster infection), T2DM increased the risk for persistent post-zoster pain by 18% [19]. Several studies also showed that zoster-related pain is more intense in patients with T2DM [34]. Impaired glucose tolerance is also a risk factor for post-herpetic neuralgia [35].

In a recent retrospective population-based study, herpes zoster infection resulted in greater use of healthcare resources among patients with T2DM than in non-diabetic subjects [27]. More specifically, patients with T2DM had more outpatient visits, were prescribed more antiviral agents and had higher risk for hospitalization and longer periods of sick leave [27]. In addition, quality of life is worse in patients with T2DM following herpes zoster infection than in non-diabetic patients and improves more slowly [34]. Of note, a worsening of glycemic control has been reported in patients with T2DM who are affected by herpes zoster [27].

## Prevention

A live attenuated vaccine for the prevention of herpes zoster was first licensed in 2006 and contains the Oka VZV strain [36]. According to the Advisory Committee on Immunization Practices (ACIP), this vaccine is routinely recommended for individuals 60 years or older, including those with a previous episode of herpes zoster. Immunization is contraindicated in pregnant women, in patients with primary or acquired immunodeficiency, and in subjects with a history of anaphylactic reaction to components of the vaccine. Diabetes mellitus is not a contraindication to the vaccine. Immunization is not systematically recommended for subjects 50–59 years old. However, severe depression, comorbidities, preexisting chronic pain or difficulty to tolerate treatment medications because of hypersensitivity or interactions with other chronic medications are factors to be considered for immunization in subjects < 60 years old [36–38]. The vaccine is safe and effective. In a large randomized, double-blind, placebo-controlled trial (*n* = 38,546 subjects older than 60 years), it reduced the burden of the disease due to herpes zoster (a measure affected by the incidence, severity and duration of the associated pain and discomfort) by 61.1%, the incidence of herpes zoster by 51.3% and the incidence of post-herpetic neuralgia by 66.5% [39]. Reactions at the injection site were more frequent among vaccine recipients than in the placebo group but were generally mild [39]. However, efficacy against herpes zoster and post-herpetic neuralgia gradually declines after vaccination [40]. Moreover, the vaccine is less effective in reducing the incidence of herpes zoster in subjects older than 70 years [39].

Data regarding the safety and efficacy of this vaccine in patients with DM are scarce. In a large population-based cohort study (*n* = 766,330), the effectiveness of the vaccine did not differ between patients with diabetic nephropathy and the general population but diabetic patients without nephropathy were not evaluated [41]. In a small study (*n* = 20 subjects 60–70 years-old), a live attenuated Oka VZV vaccine, which is licensed in Japan, boosted VZV-specific cell-mediated and humoral immunity to a comparable degree in diabetic patients and in healthy volunteers. None of the subjects developed herpes zoster during the 1-year follow-up period. Regarding safety, local pruritus developed in one patient in each group [42]. In another placebo-controlled study from Japan, the same VZV vaccine was co-administered with the 23-valent pneumococcal polysaccharide vaccine in 60–70-year-old patients with DM. The VZV vaccine did not enhance either humoral or cell-mediated VZV-specific immunity more than placebo [43]. Admittedly, the small study sample, the short follow-up period and the use of a different VZV vaccine are important limitations of the above-mentioned studies.

More recently, a recombinant subunit vaccine containing VZV glycoprotein E and the AS01B adjuvant system was introduced for the prevention of herpes zoster. In a large, randomized, placebo-controlled trial in 15,411 subjects 50 years or older, the overall vaccine efficacy for preventing herpes zoster was 97.2% [44]. It is worth mentioning that the vaccine efficacy did not decrease with increasing age [44]. In another large, randomized, placebo-controlled trial in 13,900 subjects 70 years or older, the overall vaccine efficacy in preventing herpes zoster was 89.8%. The vaccine was equally efficacious in subjects 70–79 years old and in those older than 80 years. The efficacy of the vaccine against post-herpetic neuralgia was 88.8% [45]. Notably, the efficacy of the vaccine did not decrease during the follow-up period of 3.7 years [45]. In both studies, the rate of serious adverse events did not differ between subjects who received the vaccine and those who received placebo [41, 42]. However, neither of these studies separately evaluated the efficacy and safety of this vaccine in patients with DM [44, 45]. The robust immunologic response to the vaccine, which appears to be independent of age, is an attractive characteristic of the adjuvanted vaccine, suggesting efficacy among older adults or other groups that may otherwise have a lower response to vaccination, including those with immunosuppression [46]. Moreover, this vaccine contains only a single VZV protein and cannot replicate, which probably makes it safer in these patients [46].

Accordingly, the ACIP recently recommended the adjuvanted herpes zoster vaccine over the live attenuated vaccine for herpes zoster prevention in immunocompetent adults 50 years of age and older [16]. Moreover, the adjuvanted herpes zoster vaccine is also recommended in patients who have already received the live attenuated vaccine [16]. Notably, equally strong humoral and cellular immune responses to this vaccine were observed in adults who were previously vaccinated with the live attenuated VZV vaccine [47]. The ACIP also states that patients older than 50 years with DM should receive the adjuvanted herpes zoster vaccine [16].

## Conclusions

DM appears to represent an important risk factor for herpes zoster. Moreover, the incidence of post-herpetic neuralgia is higher in these patients, and neuralgia is more severe and persistent. The introduction of the adjuvanted vaccine against herpes zoster appears to offer a useful tool for reducing the burden of herpes zoster in patients with DM. Given the strong association between DM and herpes zoster, these patients might have to be vaccinated in younger ages, particularly those with diabetic complications. However, there are limited data regarding the efficacy and safety of this vaccine in the diabetic population. It is also worth mentioning that the vaccination coverage for VZV, like the coverage for several other recommended vaccines, is low among diabetic patients [48]. Notably, the newer, adjuvanted herpes zoster vaccine requires two doses, with a 2- to 6-month interval between them, which might limit adherence to vaccination. Therefore, more studies are needed to clarify the safety and efficacy of herpes zoster vaccine in patients with DM and to improve their compliance to vaccination.
